# Orthopedics and Trauma in Children: Key Problems and Future Insights

**DOI:** 10.3390/children10010119

**Published:** 2023-01-06

**Authors:** Maher Ghandour, Matthias Klotz, Axel Horsch

**Affiliations:** 1 Department of Orthopedics, Heidelberg University Hospital, 69120 Heidelberg, Germany; 2 Orthopedics and Trauma Surgery, Marienkrankenhaus Soest, 59494 Soest, Germany

Orthopedic disorders among children are frequently encountered in clinical practice. The underlying causes of such issues can widely vary, including congenital diseases, developmental disorders, or acquired problems (i.e., infectious, neuromuscular, nutritional, neoplastic, psychogenic, or traumatic origin).

This special issue featured key research on some of the major disorders that affect the pediatric population, providing valuable insights into their diagnosis, treatment, clinical and radiologic outcomes, and prognosis predictors. The majority of articles reported original data on orthopedic issues and traumatic events that pediatric patients face in clinical settings, including developmental dysplasia of the hip (DDH), equinus foot deformity, idiopathic scoliosis, Ewing sarcoma, and bony fractures. 

DDH, one of the most common skeletal abnormalities that affect children [1], was thoroughly investigated in this issue, with research reporting the clinical and radiologic outcomes following different therapeutic options, such as foam splint [2], Spica cast [2,3], or femoral and ileum osteotomy [4]. In addition, a systematic review highlighted some of the key prognostic factors of such outcomes in patients with DHD undergoing Spica cast therapy [3]. 

Other research papers have focused on one of the most critical problems that children with cerebral palsy (CP) face which is equinus foot deformity. Previous research highlighted the magnitude of equinus deformity in CP cases, with a prevalence rate ranging from 71 to 99% [5,6]. However, secondary to the absence of a standardized definition/diagnostic criteria, an accurate estimation cannot be reached. In this issue, key research proposed a definition criterion for equinus foot by using a cutoff value of ≤5° dorsiflexion. The authors also tackled one of the main problems that face orthopedic surgeons which is the recurrence of equinus foot following its surgical management in CP cases. The research indicated a rate of 5–18% for recurrence, varying widely according to the surgical method, being the highest in single-event multi-level surgery and lowest following the Illizarov procedure [7]. 

Bony fractures in children cover approximately one-fourth of all the accidents and injuries they experience, with distal radius fractures being the most frequent ones [8]. Upper and lower limb injuries have accounted for 1 out of 5 fracture cases among children. Open fractures, in particular, pose a significant risk for deep infection and subsequent morbidity and mortality. In this issue, Kuhn et al. [9] highlighted that the duration between fracture occurrence and hospital presentation is a significant determinant of deep infection, with the type of fracture not being associated with infection. Most orthopedic surgeons focus on the clinical and radiologic outcomes following the surgical management of fractures in children; however, patients’ health-related quality of life (HR-QoL) is commonly neglected. Despite the very scarce data on HR-QoL, two of the published studies in this special issue assessed this outcome in children who have had fractures of the distal forearm and femur shaft and were treated surgically [10,11]. The extent of fractures correlated significantly with the resultant HR-QoL [10].

Idiopathic scoliosis, the most frequently reported 3-dimensional deformity of the spine, has a relatively low incidence. Based on a recent database analysis of records between 2011 and 2015, the incidence of idiopathic scoliosis was 0.497%, higher in females than males [12]. That being said, the management of idiopathic scoliosis poses a challenge for orthopedic surgeons. Research published in this issue denotes that 3D imaging can help reduce postoperative complications and/or reoperation rates in patients undergoing pedicle screw instrumentation [13]. Additionally, the implementation of the fixation, elongation, and de-rotation (FED) method can significantly improve most clinical parameters regardless of bone maturity or the size of scoliotic deformation [14]. Moreover, it has been shown that the extension of surgery can help predict the occurrence and longevity of acute postoperative pain in children undergoing posterior spinal fusion [15].

Ewing Sarcoma (ES), although rare, can occur among children and adults. It can originate from the bone or from the soft tissue around bones, referred to as extraosseous or extra-skeletal ES. To date, the clinical presentation, management strategies, clinical outcomes, and prognosis of extra-skeletal ES, particularly among children, have not been well-defined. The study of Ghandour et al. [16], which was published in this issue, is the first to provide valuable insights into the clinicodemographic characteristics of extra-skeletal ES in the pediatric population. That being said, future research should focus on the comparison between the adult and pediatric populations to determine any differences in their presentation patterns and clinical outcomes.

Children are a special population with very distinctive, yet widely variable, orthopedic issues and traumatic events. The presentation, management, and clinical outcomes in this population can differ from that of the adult population. Therefore, special care should be directed toward them. Therefore, articles within this special issue intend to contribute to the understanding of some of the most common orthopedic problems that face them with valuable insights into diagnosing and managing them.
